# Can quantitative MRI be used in the clinical setting to quantify the impact of intra-articular glucocorticoid injection on synovial disease activity in juvenile idiopathic arthritis?

**DOI:** 10.1186/s12969-019-0377-7

**Published:** 2019-11-21

**Authors:** Joshua L. Bennett, Amanda Wood, Nicola Smith, Ravi Mistry, Karen Allen, Sharmila Jandial, John D. Tuckett, S. Claire Gowdy, Helen E. Foster, Flora McErlane, Kieren G. Hollingsworth

**Affiliations:** 1 Paediatric Rheumatology, Great North Children’s Hospital, Newcastle upon Tyne Hospitals NHS Foundation Trust, Newcastle upon Tyne, UK; 2 Musculoskeletal Research Group, Institute of Cellular Medicine, Newcastle University, Newcastle upon Tyne, UK; 3 Paediatrics, Newcastle upon Tyne Hospitals NHS Foundation Trust, Newcastle upon Tyne, UK; 4 Radiology, Freeman Hospital, Newcastle upon Tyne Hospitals NHS Foundation Trust, Newcastle upon Tyne, UK; 5 Paediatric Radiology, British Columbia Children’s Hospital, Vancouver, Canada; 6 Newcastle Magnetic Resonance Centre, Institute of Cellular Medicine, Newcastle University, Newcastle upon Tyne, UK

**Keywords:** Juvenile idiopathic arthritis, Disease activity assessment, Quantitative MRI, Synovitis, Synovial volume, Intra-articular glucocorticoid injection, Remission

## Abstract

**Background:**

Juvenile idiopathic arthritis (JIA), the most common chronic rheumatic disease of childhood, is characterised by synovitis. Clinical assessments of synovitis are imperfect, relying on composite and indirect measures of disease activity including clinician-reported measures, patient-reported measures and blood markers. Contrast-enhanced MRI is a more sensitive synovitis assessment technique but clinical utility is currently limited by availability and inter-observer variation. Improved quantitative MRI techniques may enable future development of more stringent MRI-defined remission criteria.

The objective of this study was to determine the utility and feasibility of quantitative MRI measurement of synovial volume and vascularity in JIA before and twelve weeks after intra-articular glucocorticoid injection (IAGI) of the knee and to assess the acceptability of MRI to participating families.

**Methods:**

Children and young people with JIA and a new episode of knee synovitis requiring IAGI were recruited from the Great North Children’s Hospital in Newcastle upon Tyne. Quantitative contrast-enhanced MRI was performed prior to and twelve weeks after IAGI, in addition to standard clinical assessment tools, including the three-variable clinical juvenile arthritis disease activity score (cJADAS) and active joint count.

**Results:**

Eleven young people (5 male, median age 13 years, range 7–16) with JIA knee flare were recruited and 10 completed follow-up assessment. Following IAGI, the median (interquartile range) cJADAS improved from 8.5 (2.7) to 1.6 (3.9), whilst the median synovial volume improved from 38.5cm^3^ (82.1cm^3^) to 0.0cm^3^ (0.2cm^3^). Six patients presented with frank synovitis outside normal limits on routine MRI reporting. A further three had baseline MRI reports within normal limits but the quantitative measurements identified measurable synovial uptake. Post-IAGI quantitative measurements highlighted significant improvements in 9 patients.

**Conclusions:**

IAGI led to a marked reduction in synovial volume, with quantitative MRI identifying more patients with an improved synovial volume than routine qualitative clinical reporting. Improvements in cJADAS scores were more variable with the patient/parent global assessment component contributing most to the scores. Further work is indicated, exploring the utility of quantitative MRI in the assessment of less accessible joints and comparing the impact of different treatment modalities.

## Background

Juvenile idiopathic arthritis (JIA) is an umbrella term, summarising the International League of Associations for Rheumatology (ILAR) classification system for the markedly heterogeneous chronic idiopathic paediatric arthritides [1, 2]. JIA is the most common chronic rheumatic disease in children and young people with a UK prevalence of 1:1000 [3]. Affected joints are characterised by synovial proliferation and inflammatory cell infiltration, resulting in synovial effusion and hypertrophy.

JIA is a relapsing and remitting condition and the majority of young people will have more than one inflammatory flare during the two years following initial diagnosis. At least one-third of young people continue to have episodes of active inflammation during their adult years [4]. Long-standing poorly controlled synovitis is associated with a high frequency of joint damage and joint replacement surgery in adults with JIA [5]. Early aggressive therapy, including intraarticular glucocorticoid injection (IAGI) and prompt introduction of systemic immunosuppression, is thought to improve remission rates, prevent joint damage and improve functional outcomes in JIA [6]. IAGI induces rapid suppression of inflammation in targeted joints through a complex combination of effects including reduced leucocyte cell infiltration, altered leucocyte activity, reduced synovial perfusion and reduced vascular permeability [7, 8].

The heterogeneous nature of JIA and normal developmental changes of childhood ensure that no single clinical assessment can reliably capture overall disease activity in all young people with JIA [9]. At the present time, clinicians rely on composite measures of disease activity, comprising multiple indirect clinical and blood markers, including the active and limited joint counts (AJC / LJC), disability score (CHAQ) [10], ESR/CRP and global assessments by clinicians and parents. None of these accurately reflects the synovial state, and many are subjective in nature with limited repeatability [11]. JIA-specific composite tools have been used to derive multiple definitions of remission and it is not yet clear which, if any, constitutes the optimal definition [12]. The lack of a single ‘gold standard’ treatment target is one of the central barriers to implementing modern treat-to-target regimes in clinical practice [13].

Newer imaging modalities, such as musculoskeletal ultrasound (MSUS) and magnetic resonance imaging (MRI), have the potential to further inform our understanding of remission in JIA. Comparison of clinical and imaging-based definitions of remission is considered a high priority for future research [14].

Ultrasound (US) and colour Doppler techniques can detect subclinical synovitis in young people with JIA, and there have been extensive efforts in recent years to define the normal US appearance of paediatric joints [15], to create definitions for the sonographic features of synovitis in young people [16], and to standardise acquisition protocols and scoring systems [17]. Although this work is important, paediatric MSUS requires specialist skills and training and may not be available at all centres. Furthermore, the non-uniform accessibility of many joint spaces limits MSUS scoring to broad grades [18].

Contrast-enhanced MRI is able to visualise the synovitis that drives the degradative disease process, even at low levels, and is recognised to be the most sensitive technique for the assessment of synovitis [19–21]. Post-contrast T1-weighted images clearly differentiate the extent of synovial hypertrophy vs. free fluid in the joint. In fact, MRI can identify synovial changes indicative of active disease in young people with JIA who fulfil clinical criteria for inactive disease [22]. Unenhanced MRI can uniquely detect bone oedema in addition to bone erosions. However, the clinical utility of MRI has been modest due to availability and the acceptability of MRI scanning in paediatric populations. While there has been research activity demonstrating the potential of MRI in the assessment of intra-articular disease activity, there are limited normative paediatric data, recruited cohorts have had mixed presentations and there is a lack of JIA-specific clinical assessment tools to validate the findings. Consequently, there is wide inter-observer variation in the interpretation and reporting of MRI scans.

In clinical practice, synovial enhancement is reported as either being within or exceeding normal limits, based on an expected normal appearance. In research, the qualitative JAMRIS score (juvenile arthritis MRI scoring system) has been proposed as a classification of synovial hypertrophy on a 3-point scale (< 2 mm, 2-4 mm or > 4 mm thickness) in six anatomical regions but may be insensitive to longitudinal change [23, 24]. By contrast, we can seek to quantify properties of disease activity on continuous scales by image processing, rather than qualitative description or grading. The potential advantage of such an approach is the ability to discriminate change more finely than qualitative scales, and to measure disease activity that may fall below present qualitative reporting standards [25–27]. The cost of such approaches is the analysis effort required.

In summary, quantitative MRI may have considerable utility in both clinical research and the clinical care of young people with JIA, since it can provide sensitive, continuous measurement of synovial volume and the vascularity of the synovium through dynamic measurement of the uptake of gadolinium contrast agent. This study investigates whether quantitative MRI can provide a sensitive measurement of the effects of IAGI in young people with JIA, and compares synovial volume and contrast uptake measurements. The study also assesses the feasibility and acceptability of the imaging for children and parents through feedback by questionnaire and telephone interviews.

## Methods

### Subjects

Young people were recruited from a tertiary paediatric rheumatology service in the North of England (The Great North Children’s Hospital at Newcastle upon Tyne Hospitals NHS Foundation Trust). Patients and their families attending clinic were invited to consider participating in the study if all inclusion criteria were present (new or known diagnosis of JIA, new presentation of knee synovitis requiring intra-articular glucocorticoid injection (IAGI) and age 4–16 years). Exclusion criteria were steroid injection of involved knee in the past six months, concurrent use of oral or IV steroids, contraindications to contrast agent or non-sedated MRI, non-English speaking families, or recent trauma to the knee. No changes were made to concomitant immunosuppressive treatments during the study period. Families of young people who were eligible and interested at their clinic visit were followed up by telephone and invited to take part in the study.

Additional file 1: Fig. S1 shows the recruitment flow chart for the study. The subjects had two MRI and clinical assessments, the first prior to the intra-articular injection and the second at least twelve weeks after the injection. Affected knees were injected with 1 mg/kg triamcinolone hexacetonide to a maximum of 40 or 60 mg depending on weight: image guidance was not used, as per local protocol. Where both knees were affected, both were injected, and the knee judged most affected at clinical examination was followed by MRI. The study, performed between September 2017 and August 2018, complied with the Declaration of Helsinki and obtained a favourable opinion from the Newcastle and North Tyneside 1 Research Ethics Committee and the Health Research Authority, with the parents/carers of the subjects giving written informed consent, and written assent from the young people. Routine clinical care was not altered by the addition of the MRI scanning. KGH had control of the study data and had responsibility for overseeing data acquisition and processing.

### Clinical assessments

Clinical assessment was performed at baseline and follow-up. This included measurement of the core outcome variables: binary 74-joint active and limited joint counts, physician global score of disease activity (10 cm visual analogue scale (VAS)), patient/parent global score of disease activity (10 cm VAS), CHAQ and ESR/CRP (where available) [28], a global pain score (10 cm VAS) and the 3 and 4 variable juvenile arthritis disease activity scores (cJADAS and JADAS) [29, 30]. Clinical remission was defined according to the JADAS and cJADAS cut-offs for inactive disease (</= 1) [31, 32].

### MRI protocol

A peripheral venous cannula was inserted in the upper limb. Subjects were made comfortable in a supine feet-first orientation on a Siemens 1.5 T Espree, using an extremity birdcage coil. The MRI sequences (including T2-, T1- and proton density weighted scans, Additional file 1: Table S1) were prescribed so that the bottom slice passed through the proximal aspect of the superior tibiofibular joint. The leg position and anatomical location were carefully matched at the post-treatment visit. The sequences included pre- and post-contrast T1-weighted (T1w) imaging to distinguish synovial enhancement and a multiple dynamic T1w sequence was used to collect dynamic gadolinium uptake data before, during and after the injection of contrast agent. After two dynamics (26 s), the contrast agent was administered by hand as a standard single dose of gadoterate meglumine (Dotarem, 0.2 ml/kg body weight, Guerbet), followed by a 3 ml flush of 0.9% sodium chloride. The uptake of contrast was imaged for five minutes post-injection. The post-contrast T1w fat suppressed turbo spin echo imaging was collected immediately after the dynamic series ended such that post-contrast data were collected 5 min post injection as recommended [33].

### Image processing

**Fig. 1:**
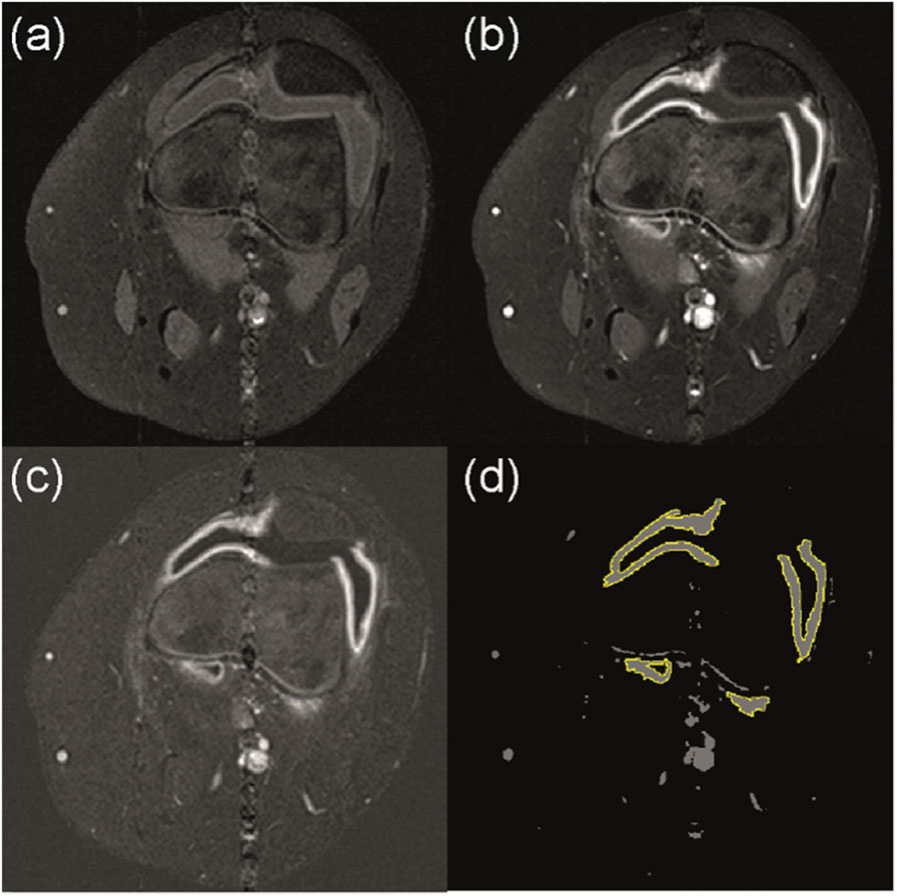
Procedure for calculating the synovial volume, shown on one slice of the imaged knee volume. The acquired data are (**a**) the multi-slice T1-weighted FS TSE pre-contrast and (**b**) post-contrast showing synovial hypertrophy. (**c**) These image stacks are subtracted to produce a stack of difference images which highlight the signal change caused by contrast uptake compared to other tissues; (**d**) thresholding these values helps to segment the enhancing synovium from structures with low uptake, though blood vessels remain visible. The magic wand tool of ImageJ is used to select the enhancing synovium; the volume is calculated by summing across the image stack. FS TSE, fat saturated turbo spin echo

**Fig. 2:**
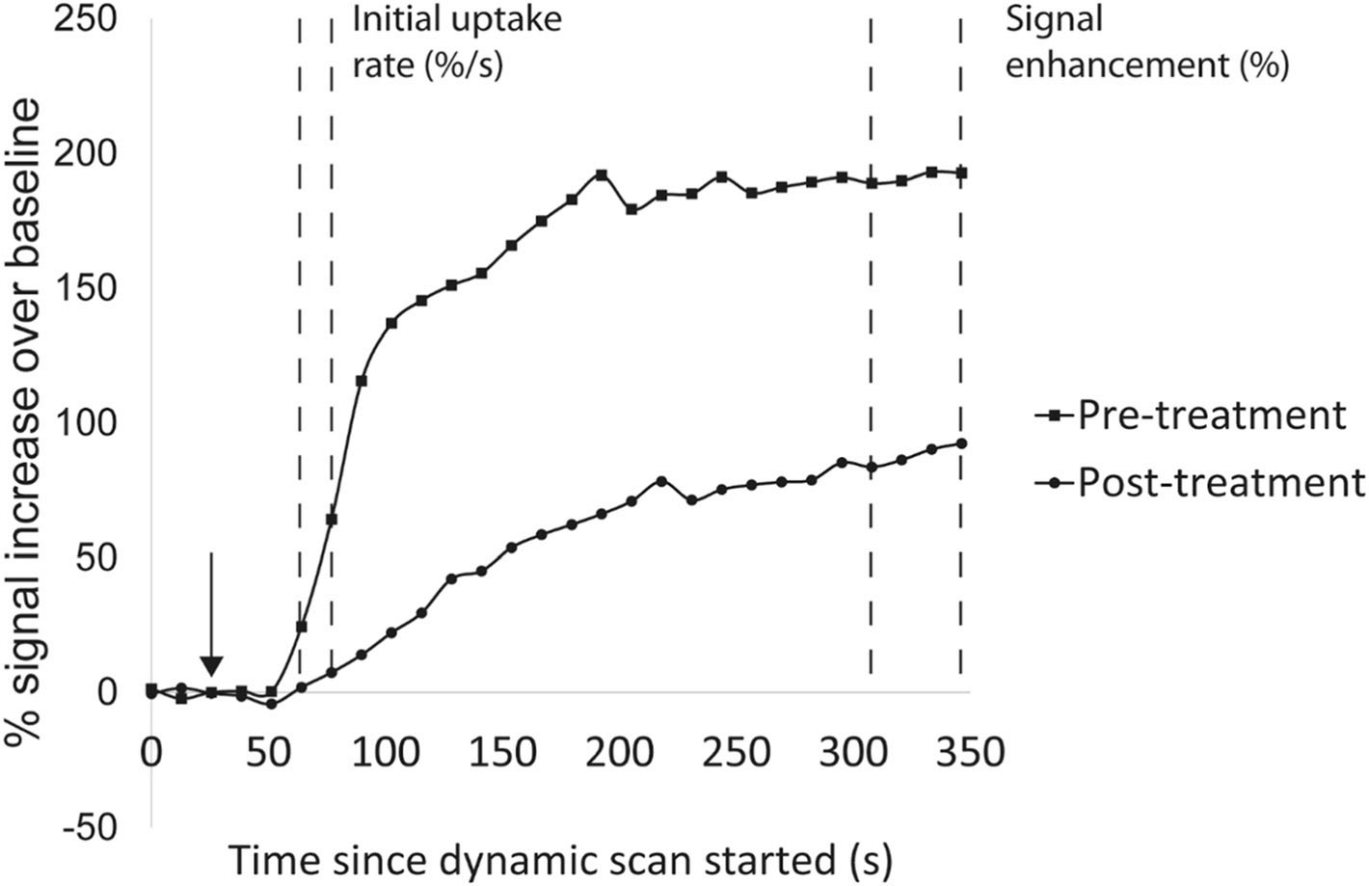
Percentage gadolinium signal increase across the segmented synovial volume for subject 5. The contrast is injected at the time shown in the arrow. The initial uptake rate (%/s) is calculated from the gradient of the line of the first two positive signal increases and the signal enhancement (%) is the mean of the last four points. This subject had a change in synovial volume from 72.2cm^3^ pre-treatment to 0.7cm^3^ post-treatment. The initial uptake shown reduces from 2.83%/s to 0.51%/s post-treatment and signal enhancement reduces from 190 to 90%

The MRI images were reported by a consultant radiologist (JDT) and were also quantitatively processed using custom software in MATLAB 2017a (Mathworks, UK) and ImageJ v1.43u (NIH, USA). The images of the dynamic gradient echo sequence and the post-contrast T1w sequence were registered to the pre-contrast T1w sequence to eliminate the effect of minor subject movement during the scan. A difference image stack was formed by subtracting the image intensity of the pre-contrast T1w sequence (Fig. 1a) from the post-contrast (Fig. 1b) to highlight the intensity of gadolinium uptake (Fig. 1c). This difference image was thresholded and binary gated to allow semi-automated delineation of the enhancing synovium from adjacent tissues throughout the imaging volume (Fig. 1d): the synovial volume was calculated from this by automatically summing the areas from each slice and multiplying by the slice thickness (see Additional file 1: for further detail). The signal increase due to contrast agent entering the synovium was expressed as a percentage increase compared to the first four dynamics before the gadolinium arrived (Fig. 2). Using the delineation of the synovium, the initial rate of uptake was estimated (in %/s) from the gradient of the first two measurements after the arrival of contrast (Fig. 2). The signal enhancement (in %) was calculated as the mean percentage increase of the final four dynamics of the series (Fig. 2, see Additional file 1 for further detail). Quantitative analysis was performed by two observers working independently (KGH, an MRI physicist with 12 years’ experience in MSK imaging, and JLB, a specialist trainee in paediatrics).

### Patient acceptability questionnaires and telephone feedback

To assess the acceptability of our research protocol, we asked the families to provide feedback in two ways. At the end of the first MRI appointment, young people and their families were invited to complete a NHS England ‘Friends and Family’ questionnaire [34] and this was followed up with a telephone interview in which the families were asked to discuss their experience of the MRI visit in more detail. The topics presented in this telephone interview and the formal analysis methods used are given in Additional file 1.

### Time occupation of the scanner suite, and study subject time

For all MRI sessions, scan timings were extracted from the DICOM headers. Where feasible, the total time between the families arriving and leaving the MRI unit was measured.

### Statistical analysis

Data were analysed in SPSS version 24 (RSI, Boulder) and expressed as median (interquartile range, IQR) except where medians and ranges have been explicitly stated. The changes in synovial volume, initial rate of contrast uptake and signal enhancement between the baseline and follow-up visits, AJC, cJADAS and pain global score were analysed with the non-parametric Wilcoxon signed-rank test. The inter-observer agreement was assessed by using Bland-Altman analysis [35]. The bias and 95% limits of agreement was calculated between the two observers. Association between synovial volumes and clinical assessments was performed using the Spearman’s rank test.

## Results

Eleven young people (5 male; 6 female; median age 13.4 years; age range 7.7–16.0) participated successfully in the baseline studies. Demographic and clinical data (including concomitant medication) are presented in Table 1. Ten young people attended and successfully completed the second MRI research visits: contact with one family was lost and follow-up data could not be collected. Only one family approached with study information declined to take part in the research.

**Table 1:** Patient characteristics and summary of clinical assessments at baseline (B) and follow-up (F)

Subject	Age	Gender	Disease duration (months)	ILAR Subtype & Systemic Medication	Active joint count		Clinician assessed disease activity in injected knee (Y/N)		JADAS10		cJADAS10		ESR		CHAQ		Pain global score (cm)	
					B	F	B	F	B	F	B	F	B	F	B	F	B	F
1	12.3	F	0	psoriatic ^m^	1^lk^	1^lk^	Y	Y	n/a	5.5	9.3	5.5	n/a	10	1.4	n/a	6.1	4.0
2	7.7	F	27	per oligo ^m^	2^rk,ra^	–	Y	–	7.0	–	7.0	–	20	–	0.3	–	3.2	–
3	16.0	F	8	per oligo ^z^	2^lk,rk^	0	Y	N	8.4	0.0	7.8	0.0	26	16	0.5	0	3.3	0.0
4	11.6	M	140	per oligo	1^rk^	1^rk^	Y	Y	4.2	2.0	4.2	2.0	5	2	1.0	0	3.6	0.0
5	13.4	M	53	ext oligo	1^lk^	1^rk^	Y	N	7.1	n/a	7.1	3.0	2	n/a	0.0	0	3.0	1.5
6	16.2	F	6	RF+ poly ^m,d^	2^lk,rk^	0	Y	N	7.2	0.0	7.2	0.0	5	5	1.0	0.3	2.9	0.9
7	13.6	F	54	per oligo ^d^	2^lk,rk^	0	Y	N	12.4	0.0	11.0	0.0	34	15	0.5	0	6.3	0.0
8	8.1	M	0	per oligo	2^lk,rk^	2^rk,ra^	Y	Y	18.8	8.9	15.6	8.9	52	9	1.9	1.9	10.0	6.8
9	13.4	M	74	ext oligo ^d^	2^lk,rk^	0	Y	N	10.8	1.2	10.0	1.2	28	15	1.3	n/a	6.5	6.1
10	15.3	F	92	RF- poly	1^rk^	0	Y	N	9.2	n/a	9.2	4.2	16	n/a	1.3	n/a	7.1	3.4
11	14.3	M	14	per oligo	1^rk^	0	Y	N	2.0	0.0	2.0	0.0	2	5	0.0	n/a	0.0	0.0
Median	13.4		27		1.5	0.0†			–	–	8.5	1.6*	–	–	–	–	4.9	1.2^$^
IQR	2.8		57		1.0	1.0			–	–	2.7	3.9	–	–	–	–	3.4	3.9

Medians and interquartile ranges (IQR) comparing baseline and follow-up exclude subject 2 who was lost to follow-up. Where measurements are not available for other individuals, medians have not been provided. †*p* < 0.03, * *p* = 0.005, ^$^ *p* = 0.008 at follow-up compared to baseline. Key: per/ext. oligo = persistent/extended oligoarticular, RF+/RF- poly = rheumatoid factor positive/negative polyarticular, JADAS10 = juvenile arthritis disease activity score (10 joints), cJADAS10 = clinical juvenile arthritis disease activity score (10 joints), ESR = erythrocyte sedimentation rate, CHAQ = child health assessment questionnaire, n/a = not available. For active joint counts, superscripts specify joints (lk = left knee, rk = right knee, ra = right ankle). The imaged knee is underlined. Systemic medication: m = methotrexate, z = azathioprine, d = adalimumab

The median time between initial clinic visit and first MRI assessment was 7 days (range 6–8 days). IAGI was usually performed later the same day (median 0 days after initial MRI, range 0–21 days). The median time from joint injection to the second MRI and clinical research assessment was 14 weeks (range 12.6–19 weeks). The time required for subjects’ MRI appointments is presented in the Additional file 1.

The median time (IQR) for contrast to reach the synovium after the beginning of the scan was 63 (14) seconds and contrast never reached synovium before the fifth image (range fifth-seventh image). Therefore, the uptake of gadolinium was followed for a median time of 315 (14) seconds after injection.

### Clinical assessments

**Fig. 3:**
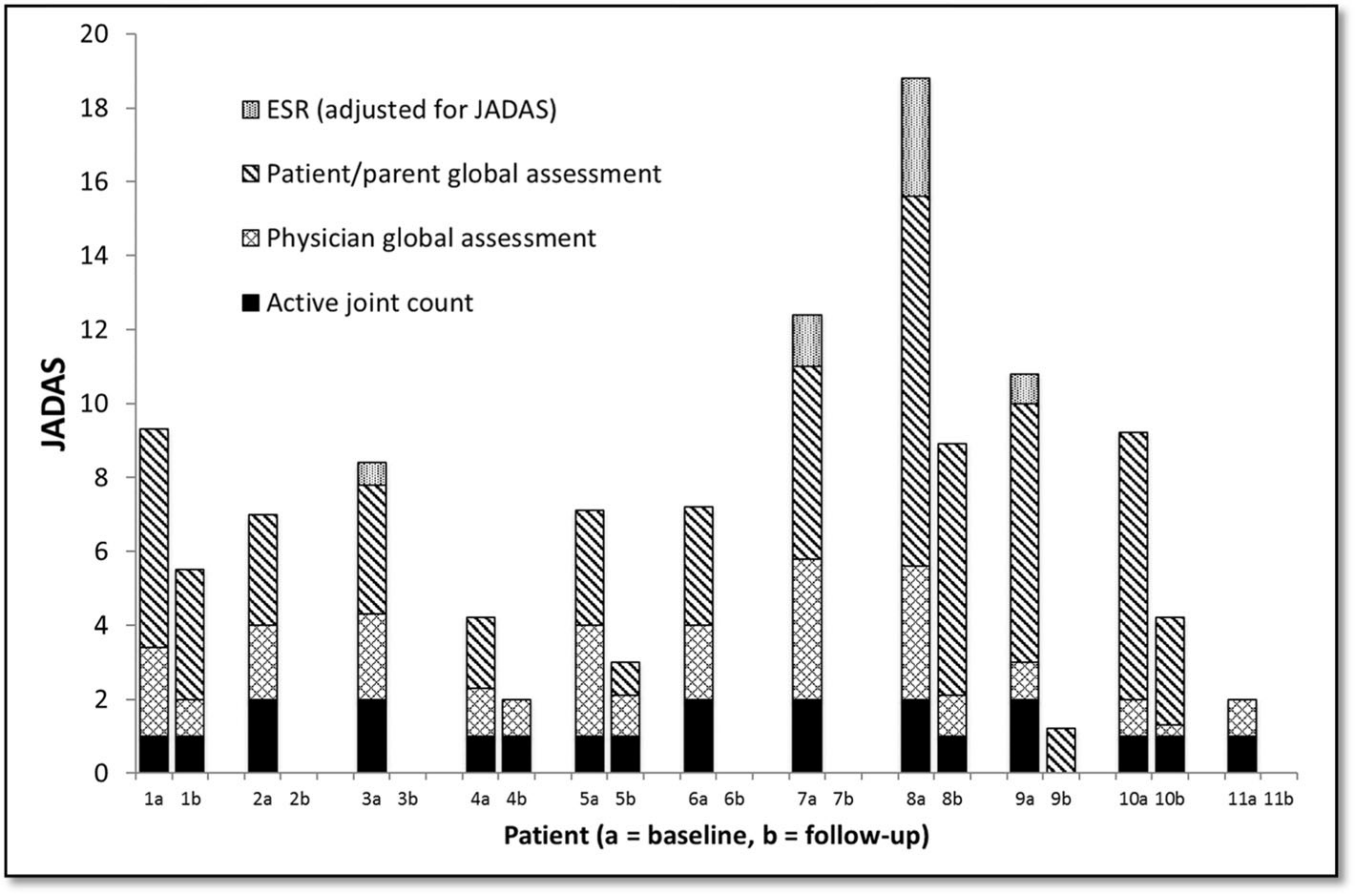
The JADAS scores for the subjects at baseline and follow-up MRI scanning. Column shading indicates the relative contribution of the four JADAS components. The overall score is often dominated by the patient/parent global assessment, with no clear relationship to the physician assessment. There was no follow-up data available for subject 2, and blood tests were not clinically indicated for subject 1 at baseline and subjects 5 and 10 at follow-up. JADAS, juvenile arthritis disease activity score; IAGI, intra-articular glucocorticoid injection; ESR, erythrocyte sedimentation rate

Baseline and follow-up clinical assessments are presented in Table 1 and Fig. 3. Clinician-assessed disease activity in the injected knee changed from present to absent in 7/10 participants post-IAGI. Two of the three participants with persisting clinician-assessed disease activity had a measurable synovial volume on follow-up MRI; one did not. All three participants with persisting clinically-assessed disease activity had repeat IAGI. This subsequently led to clinical improvement in the two with a measurable synovial volume and no improvement in the third young person. Although the AJC decreased significantly overall, it did not change in 4/10 patients. The physician global score reduced after joint injection in all cases, as did the parent global score for 9/10 cases. Where the ESR was > 5 mm at baseline, it always decreased. 6/10 patients had an AJC of 0 following joint injection and 4 of these 6 patients had follow-up JADAS and cJADAS scores of less than or equal to 1 (consistent with inactive disease or remission): no other patients had JADAS/cJADAS scores indicating remission. Figure 3 illustrates that the parent global scores generally contributed more to the overall JADAS score than the AJC or physician global score with no suggestion of a relationship to ILAR subtype or disease duration.

### Qualitative MRI reports

For the first MRI assessment, synovial thickening outside normal limits was reported for six of the subjects at baseline and reported within normal limits for five subjects (Tables 1 and 2). Minimal synovial enhancement in keeping within normal limits was noted in the report on two of the latter subjects, and no enhancement noted on the other three subjects. All reports for the second MRI assessment found no synovial thickening beyond normal limits. Chondral surfaces all appeared to be well maintained and there was no evidence of bone oedema.

**Table 2:** Quantitative MRI results at baseline (B) and follow-up (F)

Subject	Synovial volume (cm^3^)		Maximum uptake rate (%/s)		Signal enhancement (%)		Radiologist reports synovial enhancement beyond normal limits (Y/N)		Clinician assessed disease activity in injected knee (Y /N)	
	B	F	B	F	B	F	B	F	B	F
1	25.7	0.0	1.5	0.0	116	0	Y	N	Y	Y (rpt IAGI)
2	0.6	–	0.5	–	35	–	N	–	Y	–
3	1.2	0.0	0.4	0.0	57	0	N	N	Y	N
4	51.2	0.1	1.8	0.4	148	109	Y	N	Y	Y (rpt IAGI)
5	72.2	0.7	2.8	0.5	190	90	Y	N	Y	N
6	0.1	0.0	0.0	0.0	10	6	N	N	Y	N
7	92.2	0.0	6.7	0.0	225	0	Y	N	Y	N
8	101.6	1.9	4.6	1.9	157	126	Y	N	Y	Y (rpt IAGI)
9	87.0	0.0	7.1	0.0	228	0	Y	N	Y	N
10	1.2	0.0	0.6	0.0	95	0	N	N	Y	N
11	1.1	0.2	1.5	0.8	145	103	N	N	Y	N
Median	38.5	0.0†	1.7	0.0^$^	147	3†	–	–		
IQR	82.1	0.2	3.3	0.5	82	100	–	–		

Baseline medians and IQRs (interquartile ranges) exclude subject 2 who was lost to follow-up. † *p* = 0.005, ^$^ *p* = 0.008 at follow-up compared to baseline. Key: rpt IAGI = repeat intra-articular glucocorticoid injection after follow-up

### Quantitative MRI measurements

**Fig. 4:**
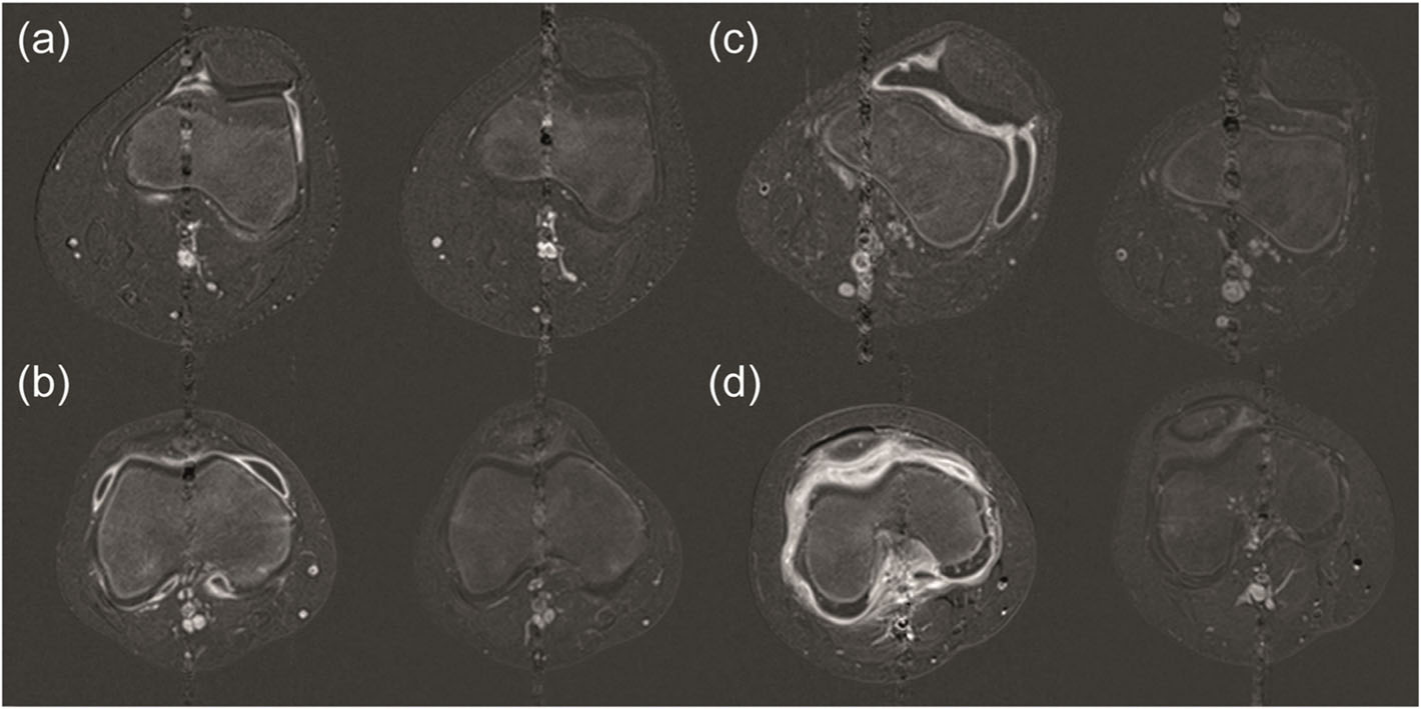
Samples of difference images comparing measured synovial volume before and after IAGI. The difference images were derived from the pre- and post-contrast T1-weighted FS TSE images to highlight the contrast uptake in the synovium for four subjects: (**a**) 12 year-old female (25.7cm^3^ vs. 0.0cm^3^), (**b**) 11 year-old male (51.2cm^3^ vs. 0.1cm^3^), (**c**) 16 year-old female (72.7cm^3^ vs. 0.7cm^3^) and (**d**) 8 year-old male (101.6cm^3^ vs. 1.9cm^3^). The whole synovial volume is quoted in each case, not just for the slice shown. FS TSE, fat saturated turbo spin echo

The synovial volumes measured at baseline and at follow-up are given in Table 2. Across the 10 subjects who attended follow-up appointments the median (IQR) synovial volume was reduced from 38.5 (82.1) cm^3^ to 0.0 (0.2) cm^3^ (*p* = 0.005). There were three signatures of synovial volume response. The first group (*n* = 6) had frank synovitis, called qualitatively by the radiologist as outside normal limits and with a median synovial volume of 79.6cm^3^ (34.5cm^3^, Fig. 4). On post-treatment follow-up, these volumes reduced to a median of 0.0cm^3^ (0.6cm^3^). The second group (*n* = 3) had a measurable synovial enhancement (with volume > 1.0 cm^3^), were called within normal limits by the radiologist and had a median synovial volume of 1.2cm^3^ (0.1cm^3^), which reduced to 0.0cm^3^ (0.1cm^3^) post treatment. The remaining subject had a negligible initial volume which was unchanged post-treatment (0.1cm^3^ vs. 0.0cm^3^) within the limits of inter-observer reproducibility.

Both the median initial rate of contrast uptake and the median signal enhancement for the 10 young people completing were significantly reduced, from 1.7%/s to 0.0%/s, *p* = 0.008, and 147 to 3%, p = 0.005 respectively (Table 2 and Additional file 1).

### Inter-observer agreement

Bland-Altman analysis for inter-observer agreement for synovial volume showed no bias between observers (− 0.04cm^3^, 95% limits of agreement 1.03cm^3^, p = n.s.), negligible compared to the median volume of 38.5cm^3^. The bias and 95% limits of agreement for the initial uptake rate of contrast were 0.01%/s and 0.23%/s respectively, while those for the signal enhancement were 2.7 and 12.9% respectively. The largest discrepancy in synovial volume between observers (1.9cm^3^) occurred in the largest synovial volume measured (101.6cm^3^). All other differences were below 0.6cm^3^. Inter-observer differences are very small compared to the dynamic range of the intervention.

### Correlation between clinical and radiological outcomes

There was a positive correlation between the pain global score and the initial synovial volume (*ρ* = 0.67, *p* < 0.04). There was no significant correlation between the decrease in synovial volume post-treatment and the change in cJADAS, the change in global pain score, the parent global score or the change in active joint count (regardless of whether the total joint count or the joint count for the studied knee was used).

### Feedback from the questionnaire and telephone interview

Feedback from the questionnaire and telephone interviews was positive; all subjects reported that they would recommend our research to their friends and families. Five core experiential themes emerged from the data enabling identification of five discrete recommendations for paediatric MRI scanning (Table 3).

**Table 3:** Themes and recommendations from the patient’s perspective. Five core themes relating to the patient experience emerged from the data and from this we identified the following recommendations for paediatric MRI

Theme 1: Clear expectations *Knowing fully what to expect relaxes both the child and their family and helps alleviate any concerns or uncertainties.*	
Recommendations: • Provide detailed information to alleviate any concerns or uncertainties both prior to the scan and on the day itself. This should include detailed directions and guidance on what will happen on the day with enough information so the parent/carer can answer any questions the child may have. • Offer the opportunity to ask questions beforehand (e.g. telephone call from clinical team prior to appointment). • Provide the opportunity to view scanner beforehand.	
Theme 2: Creation of relaxing environment *Extra touches that make families feel at home help create a relaxing environment and create a more positive experience in which to have the scan.*	
Recommendations: • Use trained paediatric staff to put both child and parent/carer at ease. Communication to be on a first name basis and whilst familiarity can add an extra layer of relaxation, it is also acceptable to be simply introduced on the day. • Use staff experienced in inserting cannulas in children to avoid extra stress that difficulties with their insertion can cause. • Give the opportunity for child to listen to music of their choice during the scan. • Give the option for parent/carer or member of staff to accompany child into scanner. • Ensure pace of appointment is not rushed and is led by the child and parent/ carer’s needs. • Care to be taken regarding scan setting wherever possible to reduce unnecessary concerns (e.g. having scan alongside cancer unit viewed as not ideal).	
Theme 3: Child centred approach *Scan experience can further be improved by adopting a child centred approach in which the child is seen as key and in control of the situation.*	
Recommendations: • Direct discussion at child. • Provide child friendly information leaflets as well as parent versions. • Give the option for toys to distract child if required by child and parent/carer. • Provide ‘completion certificate’ at the end and postcard in the post.	
Theme 4: Increased understanding of the condition *An extra layer of positivity can be added to the experience by using the scan as a way to educate the families further about the child’s condition and increase their understanding of what the scan is able to show.*	
Recommendations: • Allow the opportunity to view images after the scan alongside detailed guidance on what the images show and where possible enable patients to take a copy (e.g. photo) they can show other family members, friends or teachers at school.	
Theme 5: Linking in to current treatment plan(s) *Effective management enabling linking into current treatment plans viewed positively by families.*	
Recommendations: • Link in to current treatment plan wherever possible. For example providing the opportunity to take bloods at the same time as giving contrast seen as useful especially since many children do not like needles.	

## Discussion

We have demonstrated that quantitative MRI measurements can be used to quantify synovial disease activity in a stratified group of JIA patients undergoing IAGI. The positive subject feedback shows that it is both feasible and acceptable to collect quantitative MRI data in young people aged 7–16 years with new-onset knee synovitis.

Six subjects had synovial hypertrophy outside radiological standard limits at baseline with near-complete reduction in synovial volume at follow-up, an important observation for using quantitative MRI in the context of IAGI. Nine follow-up scans demonstrated significant reductions in synovial volume, the initial rate of contrast uptake and signal enhancement, demonstrating reduced vascularity of the remaining synovium.

In six cases with post-treatment follow-up, no enhancing synovial volume was detectable at follow-up while four subjects had a small but detectable volume. Five young people did not have synovial enhancement outside radiological reporting limits at baseline, similar to the 49% of clinically active patients who did not have synovitis according to JAMRIS classification in a qualitative cross-sectional study [36]. Van Gulik et al. reported normal MRI findings in 35% of patients with clinician-defined knee synovitis [37]. In this study, quantitative MRI was able to detect volume change post IAGI in 3/4 baseline scans falling below the standard reporting threshold. 3/10 subjects had clinician-defined disease activity at follow-up, though the MRI were reported within normal limits: there was a measurable synovial volume in two of these subjects. In retrospect, the negligible synovial volume for subject 6 suggests a non-synovial aetiology to their symptoms, despite the clinical response to IAGI.

Although the impact of IAGI on synovial volume and vascularity was frequently dramatic, the quantitative MRI changes were not always reflected in the composite clinical assessments. Of the six children with no detectable synovial volume post IAGI (0.0cm^3^), only three would be regarded as in remission according to cJADAS (</=1), whilst one child with a post-treatment cJADAS score of 0 had a measurable synovial volume. Our study adds further weight to a growing concern that the present definitions of remission may require reconsideration in light of future imaging data [14].

The post-joint injection clinical assessments illustrate that, in our cohort with low joint counts, the patient/parent global scores contribute more to the JADAS score than the AJC/physician global score. This discrepancy between family / physician perception of impact of disease has been demonstrated previously [38] and our study further highlights the risk of underestimating the impact of oligoarthritis on everyday life.

There have been previous attempts to quantify synovial volume changes with treatment over similar timescales using quantitative MRI in JIA. Workie et al. studied the 3-month response to treatment, though only four subjects received IAGI at that time point [27], with modest synovial volume reduction of 26% and 29% in the initial uptake rate: the children recruited had a median of eight active joints at baseline.

A one-year follow-up of active wrist involvement in 36 children demonstrated a 50% reduction in synovial volume, though treatment types and durations were not specified [39]. In other arthritides, such as rheumatoid arthritis, more modest changes are reported with quantitative methods [25, 39, 40].

Our study suggests that quantitative MRI scanning adds important information both to clinical assessments and to current qualitative MRI reporting. We chose to assess the impact of IAGI in new onset knee synovitis, as knee synovitis is common. However, we anticipate that quantitative MRI reporting may have more potential in the assessment of disease activity in less accessible joints and in the presence of long-standing disease, joint damage or co-existing pain syndromes.

The limitations of our study include the small sample size studied. Although the study was open to sequential eligible families with young people aged 4–16 years, only one child younger than 7 years was approached. The family declined, citing concerns that their child may not lie still. Therefore, we cannot draw conclusions about the acceptability of MRI in the youngest children. Contact with one subject was lost before follow-up scanning, though their responses to the questionnaire and telephone interview following the first MRI visit were positive. The post-IAGI synovial volumes calculated in this study suggest that the synovium of healthy knee joints has a negligible volume on quantitative contrast-enhanced imaging. There are no quantitative contrast-enhanced data on young people with healthy knees: the closest study to date examined young people with knee pain and non-inflammatory diagnoses such as hypermobility and functional disorders [42].

The use of thresholding at defined signal enhancement levels reduced subjective choice between the analysts, with the decision dominating the analysis time (typically 20–30 min per volume) being that of separating the synovium from adjacent small blood vessels. These vessels appeared to have a consistent small volume on the pre- and post- IAGI imaging, so it may be possible to evaluate the volume much faster, perhaps three minutes, with further development of the analysis software and if the inclusion of minor vessels were to be tolerated, aiding clinical adoption. Translation to clinical practice requires careful selection of the management decisions in the clinical care pathway for which quantitative MRI would be used, since MRI availability is limited. These aspects are the subject of ongoing work.

This study focuses on the impact of IAGI in new-onset knee synovitis, measured by quantitative MRI. Whilst the near-complete resolution of knee synovitis post IAGI is an interesting observation, the true value of quantitative MRI techniques may be realised in future studies exploring the impact of current treatment regimes on less accessible joints such as the hip or temporomandibular joints and / or comparing the relative efficacies of different therapeutic regimes. Quantitative MRI has particular potential in young people with oligoarticular disease in whom clinical disease activity measures may not be sensitive or specific enough to reliably identify changes in the degree of localised synovial disease.

## Conclusions

This study has demonstrated that quantitative MRI can measure the effect of IAGI on synovial volume and is accessible and feasible for children aged between 7 and 16 years, with positive feedback from families. Synovial volume was reduced markedly by IAGI, either to zero or close to zero. Quantitative MRI provided different information to traditional clinical disease activity measures and was able to identify more patients with an improved synovial volume than routine qualitative reporting. In summary, quantitative MRI has the potential to add important information to our understanding of disease outcomes in JIA.

## Supplementary information

**Additional file 1.** Supplementary Methods, Supplementary Results, Supplementary Fig. S1, Supplementary Fig. S2, Supplementary Table S1, Supplementary References.

## Data Availability

The datasets used and/or analysed during the current study are available from the corresponding author on reasonable request, subject to the requirements of the study ethical approval and Caldicott approval.

## Abbreviations

cJADAS: three variable clinical juvenile arthritis disease activity score
CRP: C-reactive protein
ESR: Erythrocyte sedimentation rate
IAGI: Intra-articular glucocorticoid injection
ILAR: International League of Associations for Rheumatology
IQR: Interquartile range
JADAS: four variable juvenile arthritis disease activity score
JAMRIS: juvenile arthritis MRI scoring system
JIA: Juvenile idiopathic arthritis
MRI: Magnetic resonance imaging
VAS: Visual analogue scale
