# Supplementary material for: Can quantitative MRI be used in the clinical setting to quantify the impact of intra-articular glucocorticoid injection on synovial disease activity in juvenile idiopathic arthritis?
Source: Pediatr Rheumatol Online J. 2019 Nov 21;17:74. doi: 10.1186/s12969-019-0377-7 (PMC6873560; doi:10.1186/s12969-019-0377-7)
Supplement: Supplementary file 1 — Additional file 1. Supplementary Methods, Supplementary Results, Supplementary Fig. S1, Supplementary Fig. S2, Supplementary Table S1, Supplementary References. [file 12969_2019_377_MOESM1_ESM.docx]

**SUPPLEMENTARY METHODS**

*Image Processing*

The processed images were analysed by two observers working independently (KGH, an MR physicist with 12 years’ experience in MSK imaging, and JLB, a specialist trainee in paediatrics). The magic wand tool of ImageJ was used to easily select those areas of thresholded signal (Figure 1d) from the imaging volume that corresponded to synovium with single clicks, and to exclude blood vessels. The areas were multiplied by the slice thickness and summed to produce a total synovial volume in cm^3^. The dynamic-contrast enhanced series was analysed as follows (Supplementary Figure 2): the first four dynamics were averaged to produce a baseline intensity for each voxel for each slice. The signal in each voxel in each dynamic was expressed as a percentage change in intensity with respect to that baseline (Supplementary Figure 2e-h). A custom macro in ImageJ applied the synovium previously segmented and calculated the volume weighted signal intensity at every time point for every slice to produce uptake curves for the synovium (main Figure 2). Two measurements were taken from these contrast uptake curves. The initial rate of uptake was estimated (in %/s) from the gradient of the first two measurements after the arrival of contrast in the synovium and the signal enhancement (in %) was calculated as the mean percentage increase of the final four dynamics of the series.

*Thresholding the T1w FS TSE difference images for synovial volume*

To avoid time-consuming and subjective manual delineation of the enhancing synovial tissue in each image slice, and to reduce inter-observer variability, we created a high-resolution difference image between the registered pre- and post- contrast image stacks. From our data, we determined a threshold that would separate the enhancing synovium from adjacent structures as cleanly as possible.

Synovial fluid and cartilage have negligible early contrast enhancement. Post-hoc analysis showed that the pre-contrast synovium and skeletal muscle had similar signal intensities on T1w TSE. By relating the signal intensity equation of the T1w TSE used (S1) and the known relaxivity of Dotarem (3.6 L mmol s^-1^) (S2) to acquisitions from phantom objects with known T_1_, this indicates a T_1_ relaxation time of approx. 1100ms, which is consistent with measurements of skeletal muscle at 1.5T and of synovium at 3.0T (S3,S4). The skeletal muscle increased in signal intensity by 20% post-contrast, while all of the enhancing synovium more than doubled in signal intensity from baseline. Therefore we binary gated the difference images by the signal difference that would represent a doubling of signal intensity from skeletal muscle. This corresponds to a contrast agent concentration of approximately 0.4mM in the synovium.

This binary gating suppresses signal from synovial fluid, cartilage and fat structures surrounding the synovium, enabling easier selection. Most signal from within bone is suppressed, though in some children significant uptake from vascular structures associated with the physis exceeded this threshold and was carried into the binary mask, though this signal is not contiguous with the synovium and can be avoided in selection. Signals from arteries, veins and smaller blood vessels also exceed the threshold but only a few are contiguous to the synovium and can be eliminated. Once the synovial selections were made, they were checked by overlaying them on the post-contrast image and were in excellent visual agreement.

*Interview guide for Parent Interviews following the first MRI visit*

Following the first MRI visit and questionnaire, telephone interviews were conducted with the parent/carer to further explore the experience of the imaging. Willingness to take part and a suitable time for calling was established during the first MRI visit. It was intended that the telephone call should be concluded within 15-30 minutes, and this was guided by the amount of feedback offered from the parent/carer. The results of the questionnaire were used to inform the interview framework and these interviews enabled further exploration of the themes derived from the questionnaire.

| **Introduction**   - Greetings, confirm identity of caller and personal introduction. Confirm follow-up call as indicated in study information sheet. Check that this is a good time to call. - Interview info: confirm the purpose of the interview and that it is a follow up from attendance at MRI centre. State the expected duration of the call. The interviewer will remind the parent/carer that the call is being recorded for later transcription but that we will preserve confidentiality of individuals in the transcript and subsequent analysis. - Confirm no implications for the NHS care received by themselves or their family if they don’t want to take part. - Enquire if any questions or concerns and respond to these appropriately. - Check if still willing to go ahead (written consent will have previously been obtained). |
| --- |

| **Feedback on the experience of the family at the MRI research unit**   - So to start can you please describe your experience at the MRI centre. (Prompt: What happened on the day?) - How was it? (Prompt: What went well? What didn’t go so well?) - Was the environment suitable? Would you say it was child friendly? (Prompt: Why) - Were you able to get there OK? And parked? - Did you have enough information to know where you were going and what you needed to do? - Were you shown the scanner beforehand? Was this beneficial? (Prompt: How was it of benefit?) - Were you provided with any information beforehand? Was this useful? - What do you think are the key things families need to know before attending the MRI appointment? - Was there any further information you wish you had been told before or during your appointment? - Were you able to look at the images after? How did you find this? Was it useful? (Prompt: Why) - In terms of the clinical team were there people you’d met before? Did that help? - Do you think seeing the clinical team during the MRI scan improved the experience for [child] (Prompt: Why? / How? What did they do that was beneficial for you?). - Do you think having someone focused on [child] during the MRI made a difference to how well they tolerated the scan (Prompt: Why?). - Did you have any concerns before attending the scan? (Prompt: Explore these). - What about [child]: did they express any concerns or worries to you? - Did you have enough information or support to help with these worries or could we have done anything more? (Prompt: Explore further information needed). - If we can just focus on [child] now: how did they cope with the experience? (Prompt: Were they looked after well?). - What did they like best, and least? Did having music played whilst in the scanner help? Was it important that it was their own music? - Is there anything that could be done to improve their experience? - When the cannula was inserted for the contrast before the MRI scan, routine blood tests can be taken at the same time so [child] does not have to come back for another needle. What do you think about this? (Prompt: Is this useful?) - Has anyone in your family had a NHS scan previously – how did this scan compare? (Prompt: What was better/worse?). - Would you tell your friends and family that the MRI centre looked after you and your child well? (Prompt Why?). - Has your child offered any feedback on the scan? (Prompt: What was positive and what was negative?) - If we were to use your experience to help tell us how we need to do things in the future – what do you think are the key things we need to think about? - What is it important for us to ensure we do? - What is it important we tell people and when would you like this information? - Is there anything else you can think of that would have made your experience better or easier? - Any other comments? |
| --- |

*Qualitative Research Methods for the Questionnaire and Telephone Interviews*

The telephone interviews were audio-recorded with the participant’s consent, transcribed (and edited to ensure anonymity of respondent), and transcripts formed the data subjected to formal analysis. The questionnaire data was analysed using descriptive statistics and free-text comments using qualitative techniques. All analysis of the qualitative data was conducted according to the standard procedures of rigorous qualitative analysis using procedures from first-generation grounded theory (coding, constant comparison, memoing), from analytic induction (deviant case analysis) and constructionist grounded theory (mapping). Data collection and analysis occurred concurrently, so that issues raised in earlier rounds of data collection could be explored in subsequent ones.

**SUPPLEMENTARY RESULTS**

*Time occupation of the scanner suite, and study subject time*

The median (IQR) MRI suite time was 27 minutes (IQR 3.5 minutes). The two longest sessions took 40 minutes: the first study performed and a second session where two pre-contrast scans were reacquired due to improve the prescription. The total length of the MR sequences was 17 minutes. The median (IQR) pause in scanning to check the cannula and prepare for injection was 5 (1) minutes and the median non-scanning time excluding injection preparation (radiographer prescription, communicating with patient) was 5.5 (2.5) minutes. The total median time from the families entering the department to leaving was successfully recorded on 13 of the 21 scan sessions and was 82 (11) minutes.

*Breakdown of the initial rate of uptake and signal enhancement results*

The initial rate of contrast uptake reduced from 1.7%/s to 0.0%/s post-treatment (p=0.008, Table 2). Group 1 with frank synovitis had median (IQR) initial uptake of 3.7%/s (4.1 %/s), which was reduced to 0.2%/s ( 0.5%/s) post-treatment. Group 2 had a median initial rate of 0.6%/s (0.5%/s) pre-treatment that reduced to 0.0%/s (1.1%/s) post-treatment. The remaining subject had no measurable uptake gradient.

The median signal enhancement was also significantly reduced for the group as a whole (pre-treatment, 147%, post-treatment 3%, p=0.005). Group 1 had median signal enhancement of 174% (66%) which reduced to 45% (104%) post-treatment. Group 2 had pre-treatment signal enhancement 95% (44%), which reduced to 0% (2%) post-treatment. The remaining subject had negligible uptake at baseline and follow-up.

**SUPPLEMENTARY FIGURE S1**


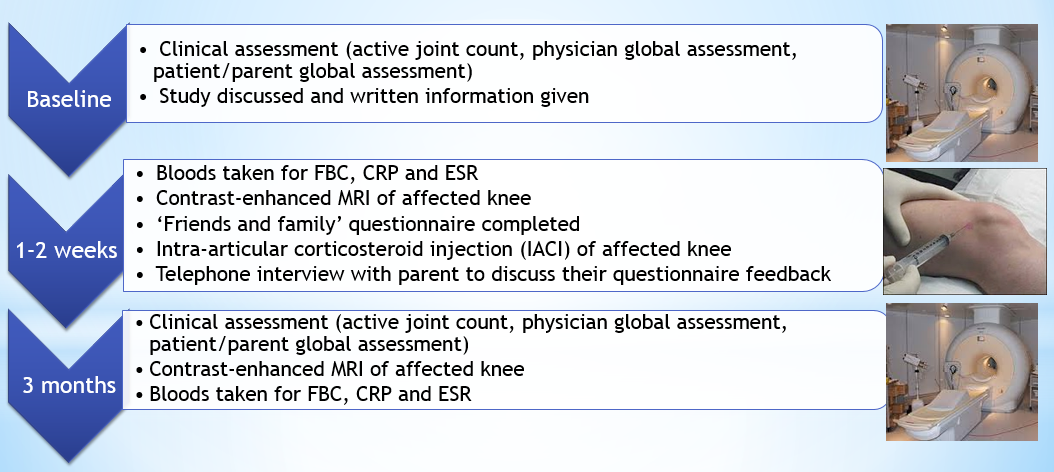
 **Supplementary Figure S1 :** Study recruitment flowchart

**SUPPLEMENTARY FIGURE S2**

**Supplementary Figure S2 :** Analysis of the dynamic 3D T1-weighted series. The top row shows the acquired images, whose signal intensities are sensitive to the concentration of contrast agent; (a) before contrast injection, (b) the first frame in which contrast enhances the synovium, (c) the last frame five minutes later. Corresponding image stacks are calculated showing percentage signal increase from baseline (e)-(g). The regions of interest defined from the volume calculations are applied (d) & (h) and volume averaged percentage signal increase is calculated for the whole synovial volume.

| *Sequence* | *Gd* | *TR/TE/NSA (ms/ms/-)* | *Flip angle (^o^)* | *Field of view (mm)* | *Voxel size (mm)* | *Slice thickness/gap (mm)* | *Recon. matrix* | *TSE factor* | *Acquisition time (min:s)* |
| --- | --- | --- | --- | --- | --- | --- | --- | --- | --- |
| Sagittal T2w FS  TSE | *-* | 4000/74/2 | 90 | 320x160x 82.5 | 0.63 | 3/0.3 | 512x358 | 10 | 3:32 |
| Axial PDw FS  TSE | *-* | 3070/21/2 | 90 | 160x160x92.4 | 0.42 | 3/0.3 | 384x384 | 6 | 3:14 |
| Axial T1w FS  TSE | *-* | 581/13/1 | 90 | 160x160x92.4 | 0.42 | 3/0.3 | 384x384 | 1 | 1:51 |
| Axial T1w 3D GRE | + | 7.4/3.7/1 | 25 | 160x160x52.8 | 0.42 | 3.3/0 | 384x384 | n/a | 13s x 28 dynamics = 6:07 |
| Axial T1w FS  TSE | + | 581/13/1 | 90 | 160x160x92.4 | 0.42 | 3/0.3 | 384x384 | 1 | 1:51 |

**SUPPLEMENTARY TABLE S1**

**Supplementary Table S1 :** Sequences used in the MRI protocol (for Siemens Espree 1.5T). FS = fat-saturated, PD = proton density, GRE = gradient recalled echo, TSE = turbo spin echo. The total sequence time is 17 minutes.

**SUPPLEMENTARY REFERENCES**

(S1) Bernstein MA, King KF, Zhou XJ. Handbook of MRI Pulse Sequences, 2004 Elsevier, p639

(S2) Rohrer M, Bauer H, Mintorovitch J, Requardt M and Weinmann HJ. Comparison of magnetic properties of MRI contrast media solutions at different magnetic field strengths. *Invest. Radiol.* 2005; 40(11): 715-24.

(S3) Gold GE, Han E, Stainsby J, Wright G, Brittain J and Beaulieu C. Musculoskeletal MRI at 3.0T: relaxation times and image contrast. *AJR* 2004; 183: 343-351.

(S4) Hodgson RJ, Barnes T, Connolly S, Eyes B, Campbell RSD and Moots R. Changes underlying the dynamic contrast-enhanced MRI response to treatment in rheumatoid arthritis. *Skeletal Radiol.* 2008; 37: 201-207.
